# Keeping the inner voice inside the head, a pilot fMRI study

**DOI:** 10.1002/brb3.2042

**Published:** 2021-01-22

**Authors:** Massoud Stephane, Mario Dzemidzic, Gihyun Yoon

**Affiliations:** ^1^ Department of Psychiatry Indiana University‐Purdue University Indianapolis Indianapolis IN USA; ^2^ Department of Neurology Indiana University‐Purdue University Indianapolis Indianapolis IN USA; ^3^ VA Connecticut Healthcare System Yale University School of Medicine West Haven CT USA

**Keywords:** fMRI, hallucinations, inner speech, Inner voice, schizophrenia

## Abstract

**Introduction:**

The inner voice is experienced during thinking in words (inner speech) and silent reading and evokes brain activity that is highly similar to that associated with external voices. Yet while the inner voice is experienced in internal space (inside the head), external voices (one's own and those of others) are experienced in external space. In this paper, we investigate the neural basis of this differential spatial localization.

**Methods:**

We used fMRI to examine the difference in brain activity between reading silently and reading aloud. As the task involved reading aloud, data were first denoised by removing independent components related to head movement. They were subsequently processed using finite impulse response basis function to address the variations of the hemodynamic response. Final analyses were carried out using permutation‐based statistics, which is appropriate for small samples. These analyses produce spatiotemporal maps of brain activity.

**Results:**

Reading silently relative to reading aloud was associated with activity of the “where” auditory pathway (Inferior parietal lobule and middle temporal gyrus), and delayed activity of the primary auditory cortex.

**Conclusions:**

These pilot data suggest that internal space localization of the inner voice depends on the same neural resources as that for external space localization of external voices—the “where” auditory pathway. We discuss the implications of these findings on the possible mechanisms of abnormal experiences of the inner voice as is the case in verbal hallucinations.

## Graphical abstract

Inner voice is a usual often unnoticeable experience associated with activities such as verbal thinking and silent reading. Abnormal experience of inner voice can lead to devastating symptoms such as verbal hallucinations and possibly other psychotic symptoms such as thought broadcasting and mind reading. Our work shows that localizing inner and external voices to internal and external space, respectively, calls upon the activity of the dorsal “where” auditory pathway.

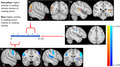

## 1 INTRODUCTION

In the expanded form, and to a lesser extent in the condensed form, of inner speech (thinking in words; Vygotsky, 1978), words are perceived as a voice—referred to as inner voice. Inner voice is also experienced during silent reading. When one reads a text or a dialogue, an inner voice is perceived as that of the self or the speakers in the dialogue, respectively. Research indicates that 97% of the population report hearing or imagining a voice during silent reading (Alderson‐Day et al., 2017). It is also possible the remaining 3% of the population, silent reading may be associated with a perceptual experience that these readers do not necessarily described as a voice. Inner voice perception appears to be intuitive and omnipresent to the point of being often unnoticeable. Neuroscience research indicates that referring to this experience as a voice is more than a figure of speech. Studies involving verbal thinking or silent reading have shown that the inner voice experience is associated with brain activity in the temporal (auditory and language) cortices (Amit et al., 2017; Perrone‐Bertolotti et al., 2012; Yao et al., 2011), including the human voice selective areas(Belin et al., 2000; Pernet et al., 2015). Inner voice appears to be processed auditorily at the brain level just like external voices.

In addition to the temporal cortex activity, inner speech and silent reading also engage the frontal motor cortex and Broca's area (Amit et al., 2017; Perrone‐Bertolotti et al., 2012). Furthermore, an extensive literature review of neuroimaging studies of language indicates that while the activity of language areas does depend on the specific linguistic operation (e.g., semantic or syntactic), language perception and expression areas activate during inner and overt speech as well as silent and aloud reading (Price, 2012).

Inner and overt types of speech are considered to evolve from a common developmental precursor (egocentric speech; Piaget, 1955; Vygotsky, 1978), and the above considerations suggest that they depend on common neural resources. However, inner speech and overt speech differ in a number of aspects (Alderson‐Day & Fernyhough, 2015), one of which is the spatial perceptual experience. Whereas overt speech is perceived in external space, inner speech is internally (inside the head) experienced. This difference in the spatial perception (inside vs. outside the head) of inner speech and overt speech is likely related to the sensory inputs associated with overt speech. However, how these inputs (or lack of) are processed at the brain level to infer internal or external space perception of speech has not been previously investigated. Although the neural mechanism of sound localization in external space is well known—the “where” auditory pathway (Romanski et al., 1999), the neural basis of the internal space experience of the inner voice remains obscure. Such knowledge is an important first step toward understanding the neural mechanisms of abnormal experiences of the inner voice as is the case in auditory verbal hallucinations (AVH)—that is, the perception of speech devoid of external speakers.

AVH are symptoms of many psychiatric and medical conditions (Stephane et al., 2015), and unlike the usual and unnoticeable experience of the inner voice, AVH experiences are unusual and often devastating to affected individuals. Decades of research suggests that AVH result from inner speech generation abnormalities (Stephane et al., 2001), and, just like inner speech, AVH are associated with activation of language perception and expression resources (Zmigrod et al., 2016). However, unlike the inner voice experience associated with inner speech, AVH are often experienced outside the head (Stephane et al., 2003). To date, the neural basis of the outside‐the‐head experience of the inner voice in AVH remains unclear.

To address the inaccessibility of inner speech to direct observation, in the present study we used functional magnetic resonance imaging (fMRI) to investigate brain activity associated with silent and aloud reading in healthy subjects as experimental models for inner and overt types of speech, respectively. As outlined above, silent reading is associated with inner voice experience as well as neural activity similar to that of inner speech. Additionally, silent and aloud readings have the same differential spatial perceptual experiences as inner speech and overt speech. We carried out model‐free spatiotemporal analyses of fMRI data similarly to our previous work (Stephane et al., 2019) after removing movement artifacts with ICA based method (Pruim et al., 2015) and employed permutation‐based statistics (Winkler et al., 2014) in our final analyses. With the above methodology, we were able to address the across subjects/tasks/brain areas variability of the hemodynamic response (HDR), movement artifacts related to reading, and the small sample in our study.

## 2 METHODS

### 2.1 Human subjects

Nine healthy subjects (5 males and 4 females, 5 Caucasian, 2 African American, and 2 Hispanic) were included in the study. All subjects were right handed, native speakers of English; and free of major medical/neurological diseases, head trauma, and active substance abuse. None had personal or family history of mental illness. Their mean age, mean personal level of education, and mean parental level of education were 42.5 ± 10, 16 ± 0.3, and 11 ± 5 years, respectively. The study protocol was approved by the institutional review board at the University of Minnesota, and all subjects gave informed consent. Furthermore, all methods were performed in accordance with the relevant guidelines and regulations. Subjects performed an internal space/external space (IS/ES) distinction task (Stephane et al., 2010) in functional magnetic resonance imaging (fMRI) environment, and all received a short task practice session before imaging.

### 2.2 IS/ES distinction task

**FIGURE 1:**
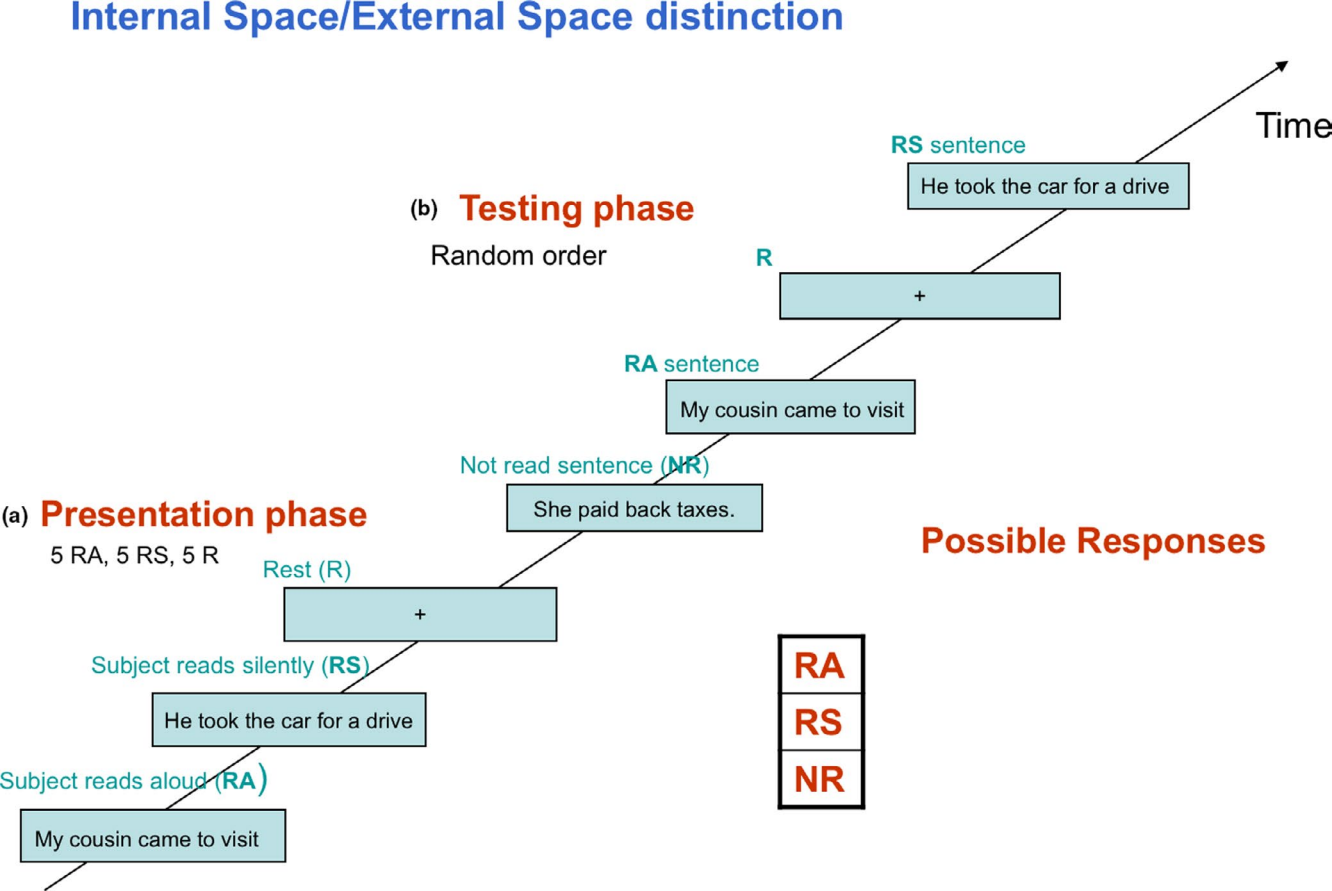
IS/ES distinction task included two phases: presentation and testing; and the presentation phase consisted of two components. In one component, subjects read aloud sentences appearing one at a time on the computer screen for a total of five sentences. In the other component, subjects read silently sentences appearing one at a time on the computer screen for a total of five sentences. These components were presented in random order. In the testing phase, the ten read sentences were mixed with five new sentences and were visually presented one at a time in a random order. During this phase, subjects were instructed to distinguish between the three types of sentences: read silently (RS) = experienced in IS, read aloud (RA) = experienced in ES, and not previously read (NR) = no space coding

The experiment was carried out in three fMRI scans about eight minutes each obtained in random order. Each scan included a presentation phase and a test phase (Figure 1). The presentation phase consisted of two components. In one component, subjects read aloud sentences that appeared on the screen one at a time for a total of five sentences. In the other component, subjects silently read sentences similarly presented. Both the components and the sentences within these components were presented in random order across scans. In the testing phase, the 10 read sentences were mixed with five new sentences and were visually presented one at a time in random order. In this phase, subjects were instructed to distinguish between the three types of sentences (read silently (RS) = experienced in IS, read aloud (RA) = experienced in ES, and not previously read (NR) = no space coding). All sentences remained on the screen for sufficient time (2 s) to allow reading at an average reading speed in the general population of 3 words/second.

The recognition of RA and RS sentences depends on IS/ES distinction while the recognition of the NR sentences reflects general recognition capacity independent of space coding. Both the presentation and testing phases included a rest period (fixation point) in equal proportion to the active events. On average, the sentences were five‐words long, had neutral affective content, and belonged to general categories such as sports and daily living. They had similar grammatical structure and were in the first, second, and third person with equal probability (Table 1).

**TABLE 1:** Sentence stimuli used in the read aloud and read silently conditions

Read aloud	Read silently
I rushed to the patient.	I appreciate my parents.
I filed a lawsuit.	I am an activist.
My office had a party.	My wife is my friend.
I hired a chef.	I bought sneakers.
I eat chocolate daily.	I eat vegetables daily.
You have a large yard.	You travel abroad soon.
Your skin burns easily.	You purchased a car.
You like diverse people.	You compete in tournaments.
You saw the president.	You opposed the war.
You work at home.	You found the basket.
He joined the discussion.	She went to the funeral.
She spent her allowance.	He held the baby.
She thanked the man.	His doctor said he was fine.
He was born in Wayzata.	She commutes to Ramsey.
He lives far away.	His basketball team won.

The test phase allowed us to ensure that the task, in particular the RS component, was carried out. If subjects do not silently read sentences as required, they would not be able to distinguish between the three types of sentences (RS, RAS, and NR). Furthermore, The task allows to disambiguate the agency of speech (self or other) from that of the spatial experience (IS or ES) of speech, both of which are shown to be independent experiences (Stephane, 2019). In the present task, reading aloud and reading silently are associated with the same agency (the self) differing only with respect to the voice spatial perception—in ES in reading aloud and IS in reading silently. Moreover, the sentences in both conditions are associated with the similar linguistic processes (such as syntax and person), which further limits the difference between the two conditions to that of the spatial perception of speech.

### 2.3 Data acquisition

Event‐related Blood Oxygenation Level‐Dependent (BOLD) response data were collected throughout the presentation and testing phases using a 3T Siemens Magnetom Trio‐Tim 3T scanner at the Center for Magnetic Resonance Research at the University of Minnesota. We used a 12 s Inter Stimulus Interval (ISI). BOLD imaging parameters were as follows: Repetition/Echo Time (TR/TE) = 2,000 ms/28 ms, Flip Angle = 80°, Field of view = 192 × 192 mm, acquisition matrix 64 × 64, 34 axial slices that were 3 mm thick with a 1 mm gap to ensure full brain view in all subjects. Therefore, for an average reading speed of 3 words per second, the sentences were read within one TR (2 s), and six BOLD volumes were obtained during each 12 s ISI allowing us to examine both the temporal and spatial dimensions of the brain activity.

### 2.4 Analyses

#### 2.4.1 Behavioral data

One‐way ANOVA was used to examine the effect of condition (RA, RS, NR) on response accuracy, and subsequent paired *t* test was used to examine the difference in response accuracy in the condition of interest (RA, RS). Furthermore, for the purpose of the fMRI data analyses, behavioral data were processed to identify the events with correct and incorrect responses for both the RA and RS sentences in the presentation phase. These analyses were carried out using SPSS 24 (IBM SPSS; Armonk, New York).

#### 2.4.2 Spatiotemporal fMRI data analyses

Recently, concerns about loss of information with model‐based analyses of fMRI data have been raised. With general linear model (GLM) (Friston et al., 1995) analyses, fMRI signals are parameterized depending on the fit between the observed data and regressors designed based on a presumed standard hemodynamic response (HDR)—that is, the temporal parameters of BOLD responses are deemed to be invariant across subjects, brain areas, and tasks. Recent evidence indicates that this assumption is less than accurate. In primates, studies point to variations in HDR across brain areas and between experimental subjects (Logothetis et al., 2001). Similarly, variations of the temporal parameters of HDR between subjects (Aguirre et al., 1998), between brain areas (Handwerker et al., 2004), according to task demands (Haller et al., 2007), and according to disease process (Dyckman et al., 2011; Ford et al., 2005; Mayer et al., 2014; Yamamotoa et al., 2014) have been reported in human subjects. Therefore, GLM‐based fMRI data parameterization can result in a loss of information about brain activity in the time dimension, and possibly the spatial dimension. Brain activity unfold necessarily in time and the above studies shows that temporal information about brain activity is relevant to physiological and pathological processes. These concerns can be addressed with model‐free analyses (Beckmann & Smith, 2004).

In this paper, we used finite impulse response (FIR) (Glover, 1999) basis functions to analyze data at the subject level. FIR basis function analyses do not presume a standard HDR function and as such are model‐free. To examine differences in brain activity between silent and aloud readings, we analyzed trials acquired during the presentation phase. Data were analyzed using FEAT tools in the FMRIB Software Library (FSL) (Oxford, England) as follows:

##### Preprocessing

Data preprocessing included removal of nonbrain tissue using BET (Smith, 2002), motion correction using MCFLIRT (Jenkinson et al., 2002), spatial smoothing using a Gaussian kernel of full width at half maximum (FWHM) of 5 mm, high‐pass temporal filtering (Gaussian‐weighted least‐squares straight line fitting, sigma = 100 s), and linear coregistration of the functional scans to the high‐resolution T1‐weighted structural scans as well as warping into MNI 151 standard space (Jenkinson et al., 2002).

##### Data denoising

As the task involved reading aloud, data were subsequently denoised using an automated classifier of head movement‐related components implemented in ICA‐AROMA software (Pruim et al., 2015).

##### First level analyses

Given the signal variability between fMRI scans, analyses were carried out separately for each scan. Using FIR basis functions, we estimated BOLD responses associated with explanatory variables that covered all event types in the task: instructions, rest (R), sentences read aloud recognized as read aloud (RA‐C), sentences RA incorrectly recognized (RA‐IC), sentences read silently correctly recognized (RS‐C), and sentences read silently incorrectly recognized (RS‐IC). Therefore, estimates of BOLD responses were obtained at 6 time points for each event (12 s ISI and 2 s TR).

#### 2.4.3 Second level analyses

These analyses were carried out using fixed effects model to compute the mean responses across runs for each subject and each event type and at each time point.

#### 2.4.4 Third level analyses

We employed permutation‐based statistics which is appropriate for the small sample size in the present study (Winkler et al., 2014). Permutation analyses have also previously shown lower percent of family‐wise errors than most parametric analyses implemented in both FSL and SPM (Eklund et al., 2016). Only events with correct responses were included in these analyses. We used one sample *t* tests to examine the contrast RS‐C/RA‐C at each of six time points and corrected for multiple comparisons using threshold‐free cluster enhancement (Smith & Nichols, 2009). Therefore, spatiotemporal maps for the difference in brain activity between silent reading and reading aloud were obtained.

## 3 RESULTS

### 3.1 Behavioral data

The one‐way ANOVA showed no significant condition effect. Nonetheless, we specifically examined the difference in response accuracy between the RA and RS conditions with paired *t* test, and similarly found no significance with 1 ± 2.3 mean difference between conditions. The means of accuracy of recognition were 12.5 ± 1 and 11.5 ± 2.6 for sentences read aloud and sentences read silently, respectively.

### 3.2 fMRI data

**FIGURE 2:**
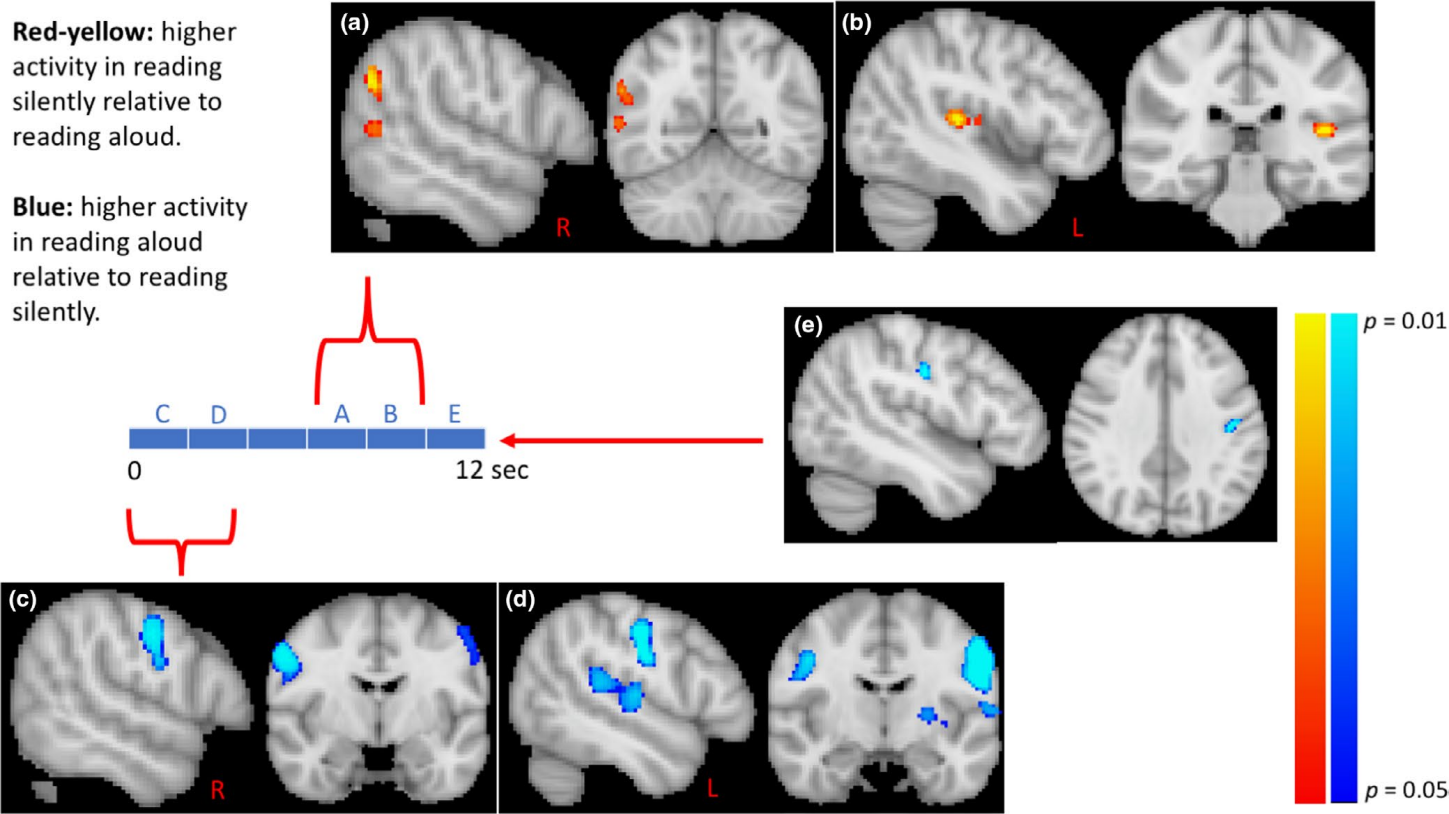
Differences in brain activity between silent and aloud readings at each time point poststimulus. Higher activity in silent reading relative to reading aloud (red‐yellow color) is observed in the inferior parietal lobule (IPL) and middle temporal gyrus (MTG) 6–8 s poststimulus (a), and in the primary auditory cortex (PAC) 8–10 s poststimulus (b). In reading aloud relative to silent reading, higher activity (blue color) was noted in the left and right primary motor cortices in the first four seconds poststimulus (c, d), and in the PAC and planum temporale (TP) 2–4 s poststimulus (d). Activity in the left PMC was also noted 10–12 s poststimulus (a)

Higher activity in silent reading relative to reading aloud was observed in the right inferior parietal lobule (IPL) (*p* < .01) and the right middle temporal gyrus (MTG) (*p* < .04) 6–8 s poststimulus, and in the left primary auditory cortex (PAC) (*p* < .01) 8–10 s poststimulus. Higher activity in reading aloud relative to silent reading was observed in the right and left primary motor cortex (PMC) (*p* < .01 and *p* < .04, respectively) in the first two seconds poststimulus, and in the 2–4 s poststimulus (*p* < .02 and *p* < .01, respectively). Higher activity was also observed in the left PAC (*p* < .02) and left planum temporale (*p* < .02) 2–4 s poststimulus, and in the left PMC 10–12 s poststimulus (*p* < .02). (Figure 2 and Table 2).

**TABLE 2:** Differences in brain activity between silent and aloud readings at each time point poststimulus

	**Time points**					
	0–2 s	2–4 s	4–6 s	6–8 s	8–10 s	10–12 s
Higher activity in silent reading				Right IPL (17, 34, 50) *p* < .01 *z* > 5.6 78 voxels	Left PAC (67, 48, 42) *p* < .01 *z* > 5.8 222 voxels	
				Right MTG (17, 34, 40) *p* < .04 *z* > 5.1 43 voxels		
Higher activity in aloud reading	Right PMC (17, 60, 53) *p* < .01 *z* > 5.4 450 voxels	Right PMC (17, 60, 48) *p* < .02 *z* > 6 99 voxels				
	Left PMC (68, 56, 53) *p* < .04 *z* > 7.3 521 voxels	Left PMC (74, 60, 48) *p* < .01 *z* > 6.3 722 voxels				Left PMC (68, 55, 53) *p* < .02 *z* > 5.6 67 voxels
		Left PAC (68, 54, 39) *p* < .02 *z* > 5.5 318 voxels				
		Left PT (69, 47, 43) *p* < .02 *z* > 5.5 318 voxels^a^				

Abbreviations: IPL, Inferior parietal lobule; MTG, middle temporal gyrus; PAC, primary auditory cortex; TP, planum temporale.

^a^ Left PAC and Left PT were combined as they are contiguous cluster.

## 4 DISCUSSION

Consistent with the literature, silent and aloud readings did not differ in associated brain activity in Wernicke's or Broca's areas highlighting a commonality in language processes at the perception and execution levels. While, not unexpectedly, reading aloud was associated with higher activity in the PMC relative to silent reading, the PAC was activated in both types of reading—an indication of a perceptual nature of the inner voice experience during silent reading.

Furthermore, we note that the PAC activity in silent reading occurred 6 s later than that in reading aloud. Perception is quasi‐instantaneous but not the related BOLD response; it unfolds over 24 s, and its temporal profile depends on the task at hand (Haller et al., 2007) (see also fMRI analyses section). Although silent reading is faster than reading aloud (Rubin, 2013), auditory cortex activity was slower in the former. We suggest that the delayed PAC activity in silent reading relative to reading aloud accounts for the different perceptual qualities (vividness) of the inner voice relative to that of external voices.

Our study findings, more importantly, suggest a neural basis for the inner space experience of the inner voice in the framework of the dual dorsal “where” and ventral “what/who” auditory pathways (Belin & Zatorre, 2000; Romanski et al., 1999). Silent reading, relative to reading aloud, was associated with higher activity in components of the “where” auditory pathway, including the right IPL (Arnott et al., 2004; Zatorre et al., 2002) and right MTG (Arnott et al., 2004; Bushara et al., 1999). These areas activate in tasks involving sound localization in external space; and, as our data show, in internal space localization of the inner voice. It is presumed that these areas perform a sort of Fourier transform to separate the external sound wave from an added filter (wave) that is dependent on the spatial location of the external sound and on the shape of the head and pinna. The inner voice associated with silent reading is devoid of a sound wave but appears to affect the primary auditory cortex similarly to a sound wave. The inner voice is also devoid of an added filter wave. However, the activation of the above areas in silent reading suggests that a “no‐filter” could be considered as a special case of a filter referring to internal space. When someone speaks aloud, there is also an added filter to his/her own voice. However, the latter filter is invariant and might serve as a default filter in a neural library of filters representing external and internal spaces.

While there are many studies that investigated inner speech, to our knowledge, this is the first study to show a neural basis for the internal space experience of the inner voice. A prior study has examined brain activity associated with external sounds delivered via headphone simulating inside and outside the head perceptions, and found higher activity in the PT—a component of the “where” auditory pathway—in outside the head relative to inside the head perceptions(Hunter et al., 2003). This finding is consistent with the literature on external sound localization but does not explain internal space perception of speech. Furthermore, simulated inner space perception of external sounds is different from inner voice. Additionally, the differences in both the experimental design (speech generation vs. perception) and analyses (model based vs. model free) render the comparability of our findings to those of the above study less than straightforward.

Our findings have important implications with respect to the mechanism of the outside‐the‐head experience of the inner voice in AVH, and possibly other psychotic experiences such as thought broadcasting and mind reading. Both the internal space experience of the inner voice and the external space experience of external voices depend on the activity of the “where” auditory pathway, and as such, dysfunction of this pathway may result in external space experience of the inner voice.

As mentioned above, AVH reflect abnormalities of inner speech generation (Stephane et al., 2001). Originally, these abnormalities were considered to be limited to agency externalization (experiencing one's own inner voice as the voice of other; Frith & Done, 1989). However, recent research suggests a more complex picture. Studies have shown that inner speech abnormalities in AVH also involve spatial externalization (hearing one's own inner voice outside the head) and that agency and spatial externalizations are independent at a phenomenological and cognitive levels and are co‐related across levels (Stephane, 2019). Therefore, these externalizations could reflect dysfunction of independent neural networks. As previously suggested (Badcock, 2010; Hunter, 2004), our data point to the “where” auditory pathway in the case of spatial externalizations. While inner speech has been often examined in hallucinations (Shergill et al., 2000), the internal space localization of the inner voice in patients with hallucinations has not been investigated. Our study suggests that this line of inquiry could clarify important aspects of the neural basis of verbal hallucinations.

## 5 CONCLUSION

Localization of inner and external voices in internal and external space, respectively, depends on the activity of the “where” auditory pathway. Therefore, dysfunction of this pathway could result in external space experience of the inner voice, which could account for the outside‐the‐head perception of inner voice as occurs in AVH and possibly other psychotic experiences such as thought broadcasting and mind reading.

It should be also noted that while the experience of the inner voice during silent reading is quasi universal, like any other subjective experience, it is unlikely to be identical among individuals (Alderson‐Day & Fernyhough, 2015). Research has shown that many developmental, cognitive and psycholinguistic factors such as age and reading speed (Fujimaki et al., 2004) influence this experience. This pilot study is not powered to address these factors. Consequently, replication of the present findings with larger number of subjects that would allow to weed out the effects of variability in the inner voice experiences and in reading speed and other cognitive factors is necessary for final conclusions. Furthermore, the investigation of this pathway in patients is also necessary for clarification of the role of the where auditory pathway in hallucinations.

## CONFLICT OF INTEREST

The authors have no conflict of interest.

## AUTHORS CONTRIBUTION

Massoud Stephane involved in design of the experiment, collection and analysis of data, and writing the manuscript. Mario Dzemidzic involved in data analysis and writing of the manuscript. Gihyun Yoon involved in data collection and writing of the manuscript.

### Peer Review

The peer review history for this article is available at https://publons.com/publon/10.1002/brb3.2042.

## Data Availability

Data will be made available to interested researchers upon request.
